# Electric Current Waveform of the Injector as a Source of Diagnostic Information

**DOI:** 10.3390/s20154151

**Published:** 2020-07-26

**Authors:** Krzysztof Więcławski, Jędrzej Mączak, Krzysztof Szczurowski

**Affiliations:** Faculty of Automotive and Construction Machinery Engineering, Warsaw University of Technology, 02-524 Warsaw, Poland; jedrzej.maczak@pw.edu.pl (J.M.); krzysztof.szczurowski@pw.edu.pl (K.S.)

**Keywords:** injector, diagnostics, inductance

## Abstract

The article discusses the method of evaluation of the fuel injector operation based on the observation of the electric current parameters, which were measured with a current transducer using the Hall effect, during the dosing process. This method relies on comparison of the electric current-related values of the examined injector with the model characteristics, which are representing the properly functioning injector. A model of the fuel injector in the form of the electric current waveform that describes the changes in the electric current and voltage during its work is presented in this article. Complex equations describing the fuel injector model under discussion account for the characteristics of the current variations, with no damage-induced modifications. Due to these, the modeled electric current/voltage waveform mirrors the real conditions. The use of a mathematical model describing the voltage–current phenomena occurring during the injector operation allows determining the actual beginning and duration of the injection. The model can also be used to develop new injector diagnostic methods that can be implemented in the engine controller (ECU).

## 1. Introduction

**Figure 1:**
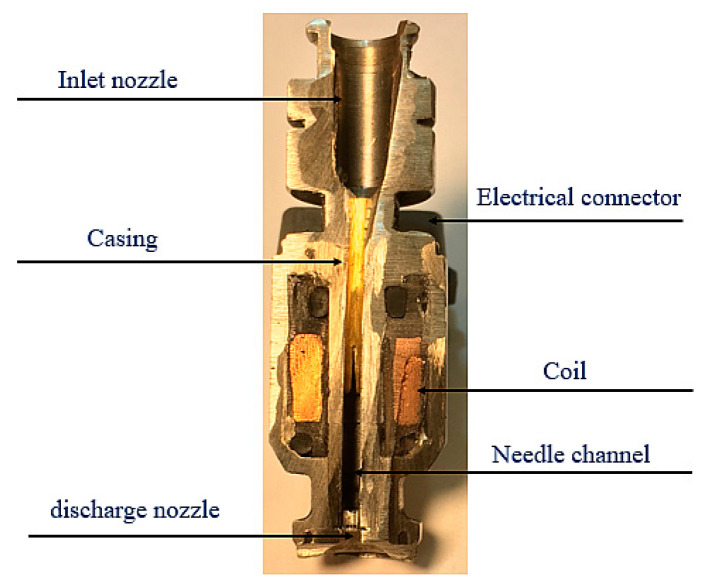
Cross-section of an electromagnetic fuel injector.

This article discusses the method of evaluation of the fuel injector operation based on the observation of the electric current parameters during the dosing process. This method relies on comparison of the electric current-related values of the examined injector with the model characteristic, representing the properly functioning injector. Figure 1 shows a cross-section of an electromagnetic fuel injector.

A model of the fuel injector in the form of the electric current waveform describing changes in electric current and voltage during its work is presented in a further section of this article. Complex equations describing the fuel injector model under discussion account for the characteristics of the current variations, with no damage-induced modifications. The developed model represents a properly functioning injector at the predetermined control parameters. 

Object modeling is widely used in diagnostics and control. Modeled are both objects and processes, where the model plays a crucial role. The fuel injector is such an assembly, playing a vital part in the supply system of a combustion engine. Modeling the control process requires developing the mechanical model, describing the needle displacements, determining the hydraulic model describing variations in the fuel flow, and developing the model of the changes in electrical values (Hung et al.) [1]. In this work, the current flow through the injector coil was described based on Kirchhoff’s law, and the basis of the mathematical model was Newton’s second law. The model built in this way based on a real object was verified in the ANSYS Maxwell and Simplorer environment. In [2], apart from determining the influence of control parameters on the resulting magnetic force, the flow of magnetic flux through the injector core was modeled. In [3,4], the simulation results were described, thanks to which the influence of the size and position of individual ferromagnetic elements on the shape of the magnetic flux, including the air gap and input voltage, was determined. Czarnigowski et al. [5] stressed that the electric current waveform can be used to compare the functioning of different injectors because it is a precise verification tool. Yao et al. [6] performed an analysis of the dynamics of the injector needle oscillation, using the mathematical model of the injector implemented in the Amesim software [7]. Factors influencing needle movement and injection quality were determined. Thanks to this, a method of optimizing the injector structure was proposed. Yan et al. [8], describing the injector dosing process, divided it into six phases, analyzing each of them and presenting theoretical corresponding calculation models. The agreement between the forecasted model and the experiment was confirmed based on current characteristics and electromagnetic parameters. (In this article, the injection phases have been divided into five ranges describing changes in current and eight ranges representing changes in electric voltage). Electromagnetic field simulations based on the injector model carried out by Wanatabe et al. [9] allowed a description of the phenomena occurring during dosing and estimation of actuator response—the needle. The magnetic field geometry was also analyzed, showing the direction of changes in the injector design in order to increase the dosing efficiency. Kay et al., in article [10], describe the influence of external factors on the method of atomizing fuel. The analysis of injection operation was carried out by dividing the injection into three main phases. Based on the fact that the quality of the injection is significantly dependent on the electric current, Xue [11] presents the optimization of the dosing process by modeling the shape of the electric current flowing through the injector coil. This resulted in the best response time of the actuator. The model includes magnetic hysteresis and the magnetostriction of ferromagnetic materials. Hung et al. [12], using differential equations, modeled the operation of the fuel injector. Thanks to the electric and mechanical model, they determined the impact of the return spring rigidity, needle mass, and number of turns of the electric coil on the characteristics of the injector, current size, electromagnetic force, and dynamics of the needle. It was proven in [13] that the use of thermodynamic models and their analysis can be used to improve the efficiency of fuel combustion and the use of energy contained in it. Kusakabe et al. [14] presented a simulation model that takes into account the magnetic resistance of the injector core, nonlinearities in its dynamics, and magnetic force. Based on the simulations, it was found that the observed nonlinearity results from the residual magnetic force. The electric current parameters were shaped, which resulted in a reduction of unfavorable oscillations of the needle.

The approaches outlined above allows for a complementary optimization of the injector’s work. In the literature, different approaches to the modeling can be found.

Modeled are the flow through the injector nozzle and the needle displacements, for the relationship between the fuel flux and the injection pressure and temperature to be determined [15,16,17]. After the characteristics have been determined, they are verified for different control parameters. Mathematical models are used to evaluate the system’s efficiency and to detect various kinds of damage. Injectors and fuel systems are modeled in special programming environments, such as Matlab/Simulink, ANSYS, or Amesim [18], due to which the system’s properties and its functioning during operation [6,19,20] are determined. Modeling enables assessing the gasoline injection and determining its most important determinants [21], as well as evaluating the influence of the design changes on the control result, i.e., the obtained engine power and quality of the combustion process. Dutka et al. [22] can detect failures in the intake system on the basis of mathematical modeling with state equations using the Kalman filter. The behavior of the needle shutting off the fuel flow is analyzed by means of computational models, which facilitates later management of the injector work [23]. Modeling that leads to the detection of damages within the system improves the reliability and safety of its functioning [24,25], by providing the tools enabling a fast diagnostics. The object-based models allow for a detailed detection of injector damages, such as blockage of the outlet channel. The process of injector control is modeled [26], and the influence of control on the solid particle emissions by the combustion engine’s exhaust system is determined [27]. The effect of the fuel composition on the efficiency of the supply system and combustion process is investigated. By means of modeling the combustion processes, the possibilities of combining different fuels are tested, which results in improving the efficiency of utilizing the energy of the fuel with a simultaneous optimization of the exhaust gas composition [28,29]. Characteristics of the differential current of the current-related waveforms are analyzed, which enables the fault detection in injectors [30,31]. Modeling is useful as far as diagnostics is concerned for performing analyses, as well as determining the performance of a working element during changes of the work parameters and control values, due to which both the diagnostics and the process of the injector control can be advanced. 

The mathematical model of an electromagnetic valve is presented in this paper, discussing changes in the values of the electric current and voltage in the coil, which are characteristic for both the parameter values and time phases.

This model is based on the exponential function, describing an increase in the current and voltage decay in the DC serial circuit in the circuit controlling the injector. The current and voltage equations have been successively formed, relating to the adequate sections of the work. Subsequently, the obtained results have been compared to the actual results obtained in the course of the laboratory experiment at the test bench mimicking the injector’s working conditions. 

## 2. Laboratory Test Stand

**Figure 2:**
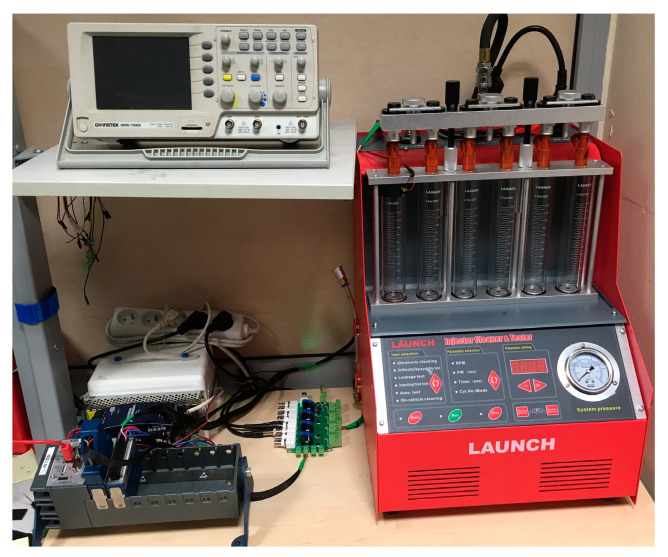
Experimental stand.

**Figure 3:**
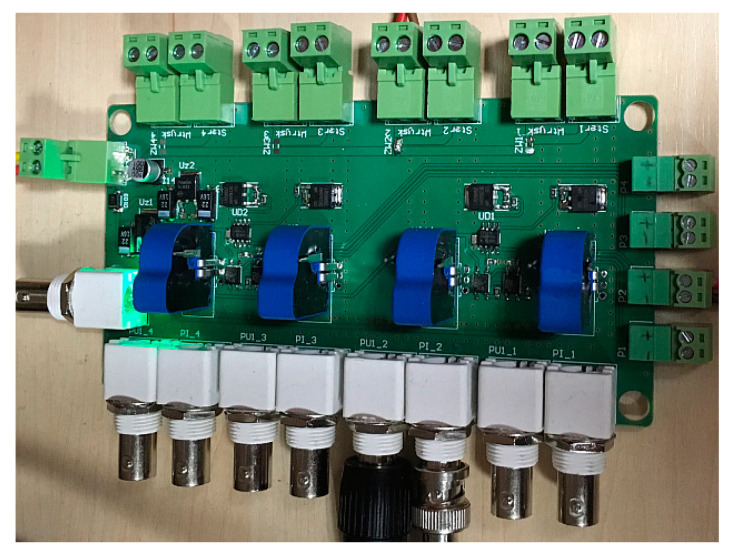
Control and measuring module.

Fuel injectors were tested on a stand dedicated for testing car injectors (Figure 2). This stand was expanded with a specially constructed computer-based measuring and control unit that managed the dosing and enabled the change of control parameters, as well as their observation and recording. This unit uses a programmable controller that controls a specially designed and manufactured for this project control and measuring module (Figure 3), which can replace the engine controller in the implementation of the injector control function and allows the observation of voltage and electric current. This allows automatization of the recording and obtaining a repeatability of measurements.

The MP-S module allows controlling the injector time parameters with microsecond accuracy. Recorded voltage and current waveforms as well as control signals are saved on the controller disk for later analysis. The system ensures a high reproducibility of results, since the beginning and duration times of the control signals are strictly controlled. In conjunction with process automation, this allows the creation of a large statistical data resource.

## 3. Fuel Injection Phases

The injector coil circuit can be considered as a serial circuit of the connected electrical resistance R and inductance L (with the electromotive force $\varepsilon_{0}$). The circuit R−L is described by Kirchhoff’s Law:

(1) $$RI+L\frac{dI}{dt}=\varepsilon_{0}.$$

Solving the following differential equation results in obtaining the equation describing the current flow (2) and voltage decay (3) in the R−L circuit (injector):

(2) $$I\left(t\right)=\frac{\varepsilon_{0}}{R}\;\left(1-exp\left(-\frac{R}{L}t\right)\right)$$

(3) $$\left|U_{L}\right|\left(t\right)=\varepsilon_{0}\left(exp\left(-\frac{R}{L}t\right)\right)$$

where exp—exponential function, I—electric current [A], $U_{L}$—electric voltage on inductance [V], L—inductance [H], R —resistance [Ω], $\varepsilon_{0}$—electromotive force [V], and t—time  [s].

Inductance of the injector core depends on the geometry of the coil core and on the magnetic permeability:

(4) $$L=\frac{\mu_{0\;\;\;}\mu_{R}\;N^{2}S}{l}$$

where N—number of turns, S—surface area of the coil cross-section $\left[m^{2}\right]$, µ—magnetic permeability of the material, $\mu_{0}$—magnetic permeability of the vacuum $4\pi \;10^{-7}$ [H/m], $\mu_{R}$—relative magnetic permeability of the material, and l —length of the coil core [m].

**Figure 4:**
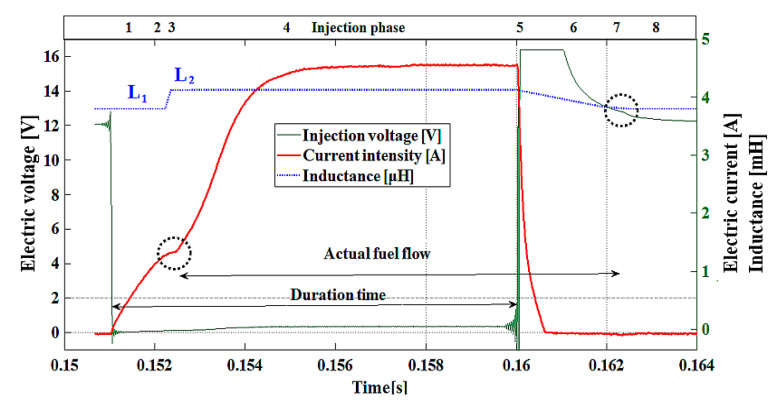
Recorded time-related waveform of injector current–voltage.

Figure 4 shows an electric current-related waveform of a dosing injector that was obtained in the course of the experiment. The current–voltage waveforms of all types of injectors are of similar character, form, and ranges. The differences are only in the scale of quantities shown in the electric current, voltage, and injection pressure waveforms.

In Figure 4, the dotted circles denote points of opening and closing the injector nozzle by the moving needle, which were determined on the basis of changes in the inductance $(L_{1},L_{2}$) and the related change of time constants $\tau_{1}$ and $\tau_{2}$.

(5) $$\tau =\frac{L}{R}$$

A change of the time constant and inductance determines ranges corresponding to the successive differential equations describing the current–voltage changes during the fuel injection. Observing the change of the derivative of the current-related waveform regarding the increase in electric current (Figure 4) enables determination of the actual needle-lifting point (from 0.1524 s to 1.526 s) with a microsecond accuracy. However, analysis of the voltage decay allows for an observation that the change in the derivative of the waveform enables ascertainment of the real point of the needle settling in the nozzle, which finishes off the flow (around 0.1622 s). As can be seen in Figure 4, the real points of opening (injection phase no. 3: dI/dt=0) and closing the flow (injection phase no. 7: dU/dt=0), are significantly delayed relative to the signal controlling the injector.

A detailed analysis of waveforms allows determining the specific phases of the injector work and the assignment of differential equations corresponding to the successive phases of the work cycle. These phases are marked in the table in Figure 4 in its top section. On the axis on the left side of this figure, there is a range of electric current shown, on the vertical axis on the right, there is a range of voltage in the coil. The horizontal axis denotes time in seconds.

Individual phases of the injector work may be identified through change in the core inductance (ΔL):

- phase 1—nozzle closed, core inductance equals $L_{1}$,
- phase 2—opening of the nozzle, change in core inductance from $L_{1}$ to $L_{2}$,
- phase 3—nozzle open, core inductance equals $L_{2}$,
- phase 4—closing of the nozzle: core inductance $L_{2}$ changes to $L_{1}$,
- phase 5—nozzle closed core inductance equals $L_{1}$.

## 4. Analysis

### 4.1. Analysis of Changes in Voltage Waveforms

Below, the successive ranges of the time-related current–voltage waveforms are discussed, together with their mathematical and physical description, and conclusions related to the possibilities of the diagnostics of the fuel injector are presented. The specific slope and position of the curve representing the model were obtained by inserting the following coefficients in the model equations:

- location coefficient ($f_{p}$),
- directional coefficient ($f_{k}$), and
- influence of pressure ($f_{press}$).

The purpose of the pressure coefficient ($f_{press}$) [32] is to obtain the modeled electric current value in the range according to the actual value. Depending on the modeled waveform segment, this coefficient takes different values. For example, for ranges 1 and 2 (Figure 4), $f_{press}$ coefficient will be less than unity, because the maximum current in that range is less than the classic Kirchhoff equation. This is the range of the characteristic bending of the electric current waveform, defining the needle rise (Figure 4). This action is opposed by fuel pressure; therefore, the current in this range is higher the higher the fuel pressure, which is associated with a higher value of the coefficient. 

The values of directional and location coefficients depend not only on electric current parameters, but also on the position in the space between the ordinate and abscissa axis of the modeled waveform section. These coefficients are a function depending on the coil resistance, inductance, electromotive force, electric current strength, and forces resisting the needle lifting. The successive waveform sections must begin at the points where the preceding sections end, so that the total waveform is continuous. A given set of coefficients is current for specific values of injector control parameters. In practice, the method of determining the coefficients is as follows: we measure the current waveform; then, knowing its characteristic points (given in Section 4), one should choose the coefficients experimentally, reflecting the shape and values of the waveforms in subsequent points.

The described model was based on the Kirchhoff equations. The Kirchhoff equation in a classic form describes the increase in current and loss of electrical voltage in the *R*−*L* circuit, which does not perform work. The current description of the injector (needle and coil of the injector) performing the work must be expanded at least by a pressure factor, thanks to which the received current at the needle lifting point will be in line with the real one (e.g., Section 1 and Section 2, Figure 4).

**Figure 5:**
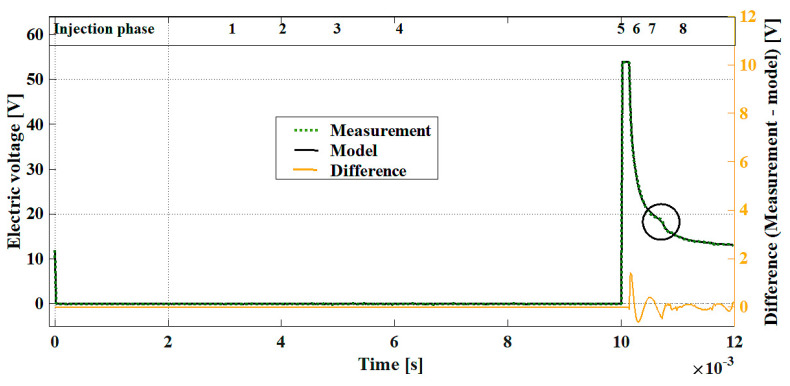
Measured and modeled voltage waveforms of the injector with the phases of differential equations marked.

- Phase 0: $U_{L,0}\;\left(t\right)$
  Before the current impulse is started $\left(t_{inj}\right)$, the voltage in the injector coil equals the supply voltage:
  (6) $$U_{L,\;0}=\varepsilon_{0}=12\;V$$
- Phases 1, 2, 3, 4: $\;U_{1,2,3,4}\;\left(t\right)$
  After initiation of the current impulse, the voltage in the coil drops from the supply voltage value $\left(\varepsilon_{0}=12\;V\right)$ to zero:
  (7) $$U_{L,1,2,3,4}\left(t_{inj}\right)=0$$
  Such a state is maintained throughout the whole injection duration. This allows for the determination of the moment when the controller starts the process of fuel injection.
- Phase 5: $\;U_{L,5}\;\left(t\right)$
  After completion of the injection, as a result of releasing the energy compensated in the coil, there occurs an inductive voltage spike, exceeding the value of the source voltage $\varepsilon_{0}$. The inductive voltage spike results from the rapid decay of the current (Section 5,  R increases to ∞; I decreases to 0):
  (8) $$U_{L,5}\left(t\right)=\frac{dI}{dt}\;L.$$
  The high value of the current derivative (rapid decay to zero) multiplied by the coil inductance causes a generation of the voltage that may be a few times greater than the source voltage.
- Phase 6: $\;U_{L,6}\;\left(t\right)$
  After the voltage spike caused by the current decay, the voltage decays exponentially, going to zero, in accordance with the equation:
  (9) $$U_{L,6}\left(t\right)=\left(f_{press}\;\varepsilon_{0}\;\left(exp\left(-\frac{R}{L_{2}\left(e^{-t}\right)}\;t\right)\right)\right)+f_{p};\;\left[L_{2}\;\mathrm{changes}\;\mathrm{to}\;L_{1};\;U_{L}\;\mathrm{changes}\;\mathrm{to}\;U_{L_{0}}\right].$$
  The injector needle pushed by the force of the spring $\left(F_{S}\right)$ and by the force resulting from the fuel pressure $\left(F_{p}\right)$, through the whole time of phase 6, shifts toward the nozzle, allowing the fuel flow. The motion of the needle being lifted due to the magnetic force is very quick. The needle returning from the coil core takes longer because the magnetic force $\left(F_{m}\right)$ is much greater than the force of the spring and of the force resulting from the fuel pressure:
  (10) $$\left(F_{S}+F_{p}\right)\ll F_{m}.$$
  As a result of the gradual lowering, the needle rests against the nozzle, which stops the flow of fuel.
- Phase 7: $\;U_{L,7}\;\left(t\right)$
  At this point, the change in the core inductance finishes in accordance with the relationship: $L_{2}\;\left(e^{-t}\right)\;\mathrm{tends}\;\mathrm{to}\;L_{1}.$ This is a transient state on the waveform of the voltage decay in the coil. The needle stoppage in the nozzle seat can be observed as a temporary (approximately 0.0002 s) flattening of the waveform of the decaying voltage:
  (11) $$U_{L,7}\left(t\right)=\left(f_{press}\;\varepsilon_{0}\;\left(exp\left(-\frac{R}{L_{1}}\;t\right)\right)\right)+f_{p};\;\left[U_{L}\;\mathrm{changes}\;\mathrm{to}\;U_{L_{0}}\right].$$
  Observation of this range in real time, through control of the derivative of the voltage waveform dU/dt, enables determination of the time of delay in closing the nozzle with the needle. The value of the voltage at which the needle settles in the nozzle determines the efficiency of the injector spring and determines the correctness of the mechanical system’s operation: the needle and the guide sleeve.
- Phase 8: $\;U_{L,8}\;\left(t\right)$
  After bringing the needle to a halt and stopping the fuel flow, the voltage decay takes place in accordance with inductance $L_{1}$, going to zero, as shown in the Equation (11). Phase 8 is terminated at a point where the voltage in the coil equals zero: $U_{L}=U_{L0}$. Figure 5 shows a modeled voltage waveform of an injector in accordance with the presented equations (6–11). The waveform shown in a dotted green line is an actual waveform. The continuous line represents the modeled voltage waveform. In the table, in the top part of the figure, the successive ranges assigned to the current equations have been marked.

In the measured waveform, the inductive spike part has been replaced with the horizontal line due to the upper limit of the measuring transducer used during the experiment. The exponential function models an increase in the electric current because the Euler’s number with the negative exponent, subtracted from one, is an asymptotic going to the set value (I(t)) with the delay dependent on the time constant. An equation with the negative, adequately big number in the exponent means a fast decrease of the quantity to zero $\left(U_{L}\right)$.

### 4.2. Analysis of Changes in Electric Current Waveforms

Below, the changes in the current waveforms are discussed considering individual injection phases.

- Phase 1: $\;I_{1}\left(t\right)$
  At the point t=0.151 s (Figure 4), a current impulse starts determined by a given injection time. At this point, the value of current I = 0. The current increases exponentially, activating the magnetic flux due to which the magnetic force $F_{m}$ is generated. At phase 1, the magnetic force $F_{m}$ is smaller than the forces counteracting the lifting of the needle $F_{0}$:
  (12) $$F_{m}<F_{0}.$$
  The injector inductance is $L_{1}$, and the time constant is $\tau_{1}$. The nozzle is closed by the needle; thus, the flow does not occur. The duration of phase 1 is about 1 ms, and the equation describing the current is as follows:
  (13) $$I_{1}\left(t\right)=\left(f_{press}\;\frac{\varepsilon_{0}}{R}\;\left(1-exp\left(-\frac{R}{L_{1}}t\right)\right)\right);\;\;\left[L=L_{1}\right].$$
  The way the current grows over time depends on the time constant τ. For example, the time constant for the coil in the *R*−*L* circuit amounts to (5). The value of the time constant can be read from the current plot for the value t=τ:
  (14) $$I\left(t\right)=\frac{\varepsilon_{0}}{R}\;\left(1-exp\left(-\frac{t}{\tau }\right)\right)=\frac{\varepsilon_{0}}{R}\;\left(1-exp\left(-1\right)\right)=0.632\frac{\varepsilon_{0}}{R}.$$
  A smaller value of the time constant means a faster increase in the electric current intensity I(t). The injector time constant (τ), determined on the basis of the resistance and coil inductance is an indicator that can be used while monitoring the electric efficiency of the coil. In the case of the injector, the time constant needs to be adopted as a specific value reached by the current after the voltage impulse has been turned on. It will determine the angle of the line denoting the increasing current intensity against the horizontal time axis (θ). The quantity I(τ) from Equation (14) cannot be applied, because it is preceded by the transient state (needle lifting) and the time constant has an altered value. The best solution is adopting a defined characteristic of the inclination of the curve denoting the increasing current intensity (angle (θ)), being a consequence of an injector’s qualities. Any change in the time constant ensues change in the increase in the current intensity at the range $\;I_{1}\left(t\right)$ and $I_{4}\;\left(t\right)$. This parameter can be monitored by observing the derivative of the current intensity at this range: dI/dt, or changing the time range from driving the injection pulse to the point of raising the needle (waveform bends =>dI/dt→0). Observation of the moment of occurrence of the whole transient state allows for evaluation of the magnetic force generated by the coil of the injector, i.e., the efficiency of its operation. Shifts in this range in time may indicate disturbances in the movement of the needle.
- Phase 2: $\;I_{2}\;\left(t\right)$
  At the point t=0.1524 s (Figure 4), there occurs a flattening of the line denoting current intensity—the beginning of the transient state. This is a point at which the magnetic flux has already generated the magnetic force $F_{m}$ of the magnitude, which counterbalanced the forces in opposition to the lifting of the needle. Equation (13) is valid here. The duration of this range is approximately 50 μs. The whole transient state lasts about 100÷200 μs (Figure 1—a black indicator, t=0.1525 s). The beginning of the transient state on the current waveform is the current intensity at the point of the needle lifting $I_{op}$. Changes in the value of the current intensity at this point indicate electrical changes in the injector coil. Shorted circuits in the coil cause a reduction in the generated magnetic flux, which in turn leads to an increase in the current. A greater current is needed for the generation of the magnetic flux, implying that the magnetic force is enough to overcome the resistance of the needle movement. Another cause for the change in the current $I_{op}$ is the changes in the fuel pressure before the injector (injection pressure). Differentiation between the discussed causes is possible based on the signal from the fuel pressure sensor.
- Phase 3: $\;I_{3}\;\left(t\right)$
  This phase takes place in the middle of the transient state. At t=0.1525 ms, the magnetic force $F_{m}$ overcame the force $F_{0}$. At this point, the process of the injector needle lifting starts. Sliding the needle into the core is synonymous with the change in the inductance of the injector coil (ΔL, Figure 4). The equation defining the current in this phase is as follows:
  (15) $$I_{3}\left(t\right)=\left(f_{press}\;\varepsilon_{0}\;\left(exp\left(-\frac{R}{L_{2}\left(1-e^{-t}\right)}t\right)\right)\right)+f_{p}\;;\;\;\left[L_{1}\;\mathrm{changes}\;\mathrm{to}\;L_{2};\frac{dI}{dt}=0\right]$$
  where $L_{2}$ is the injector inductance after lifting the needle [H], $(L_{2}>L_{1}).$
  The derivative of the waveform tends to zero, hence the denotation I(t) according to Equation (15). Due to the short time of this range, the decrease in the current is slight, but it can be observed as the flattening or bending of the line in the plot (Figure 4—black indicator (circle drawn with a dotted line)). In this phase, the fuel flow starts. In the transient state, there occurs a change in the injector core inductance, resulting from the summation of the masses of the core and the needle’s ferromagnetic material.
- Phase 4: $\;I_{4}\;\left(t\right)$
  The last phase of the transient state in the current-related waveform. The waveform of the current transitions from the decrease through the state in which the derivative of the waveform equals to zero to a rapid growth (Figure 4), which is in accordance with the equation:
  (16) $$I_{4}\left(t\right)=\left(f_{press}\;\frac{\varepsilon_{0}}{R}\;\left(1-exp\left(-\frac{R}{L_{2}}\;t\right)\right)\right)-f_{p}\;;\;\;\;\left[L=L_{2};I\;\mathrm{tends}\;\mathrm{to}\;\frac{\varepsilon }{R}\right].$$
  The final phase of the transient state overlaps with the beginning of the homogenous increase in current to the steady state (maximal for a given injection time). Two sections are combined here: the end of the transient state and an increase in the current intensity to its maximum. The maximal value at the preset duration of injection depends on the duration of this impulse. The value of the current intensity tends to quotient $\frac{\varepsilon_{0}}{R}$ asymptotically in its exponential waveform. The needle is lifted, the injector coil core has an inductance equal to $L_{2}$, and the fuel flow is continued. Resistance (R) in the Euler’s number exponent is far greater than inductance (L); e.g., the injector used in the experiment ratio R to L amounts to: 729.06 (R/L=14.8/0.0203=729.06. Therefore, the expression: $1-exp\left(-\frac{R}{L_{2}}\;t\right)\;$ has this effect in which the value of current tends to the defined value very fast.
- Phase 5: $\;I_{5}\;\left(t\right)$
  After the injection time is over, the electric current intensity rapidly decays to zero. Disconnecting the circuit means an increase in resistance (R) to infinity, and the electromotive force increases its value (inductance peak) significantly above the level of the source voltage, which is a key factor in the speed of the current decay, and it takes place in accordance with the equation:
  (17) $$I_{5}\left(t\right)=\left(f_{press}\;\varepsilon_{0}\;\left(exp\left(-\frac{f_{k}R}{L_{2}}t\right)\right)\right)+f_{p},\;\;\left[R\;\mathrm{tends}\;\mathrm{to}\;\infty =>I\;\mathrm{tends}\;\mathrm{to}\;0;\;\varepsilon ↑;\;L=L_{2};\right].$$
  This equation concludes the description of changes in the electric current at I(t)=0.

**Figure 6:**
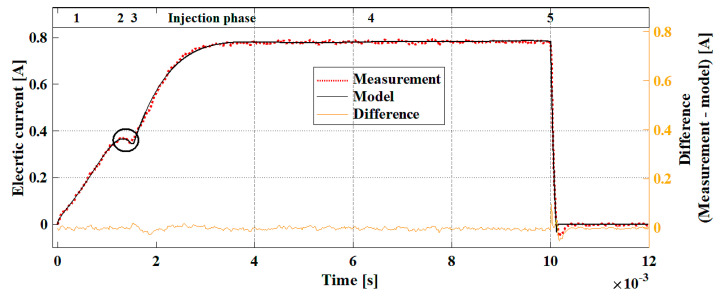
Measured and modeled electric current waveforms of the injector with the phases of differential equations marked.

Figure 6 illustrates the measured and modeled electric current waveform of the injector in accordance with presented Equations (13)–(17). The biggest differences result from the oscillations of the recorded measurement. At the time point *t =* 0.00102 s, as a result of a sudden decay of current after switching off the control impulse, the electric current drops out of range. At this point, the difference between the model and measurement is the largest (0.07 A). However, this point has no diagnostic significance, so it can be omitted.

**Figure 7:**
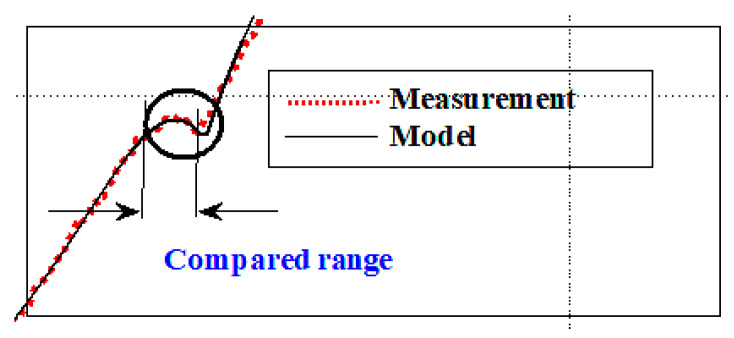
The transient section of the model and measurement for which the correlation was calculated (fragment of the graph of Figure 6).

The greatest differences are obtained for the first transient state (∆ = (0.019 A + |−0.035 A|) = 0.054 A, Figure 7). This is a satisfactory result, and the obtained model characteristics can be a reference for determining the correctness of the injector operation. Below is the calculated correlation estimator for both waveforms ($r_{pm}$) (18). The correlation was calculated for the transient range, because it is the range that is most important for early injector diagnostics. 

The linear correlation coefficient estimator is defined in the following equation (Pearson’s correlation coefficient):

(18) $$r_{pm}=\frac{\sum_{i=1}^{n}\left(p_{i}-\bar{p}\right)\left(m_{i}-\bar{m}\right)}{\sqrt{\sum_{i=1}^{n}{\left(p_{i}-\bar{p}\right)}^{2}}\sqrt{\sum_{i=1}^{n}{\left(m_{i}-\bar{m}\right)}^{2}}}\;;\;r_{pm}\in \left[-1,1\right].$$

As a result of the calculations, the correlation coefficient $r_{pm}$ = 0.9935 was obtained. The result indicates a strong fit of the model to the measurement. The maximum deviation for the value of 0.42 A (maximum transient in the current waveform) is 0.0027 A. The mathematical model of the injector can be developed with any accuracy depending on the adopted initial values (coefficients) in the equations describing it.

**Figure 8:**
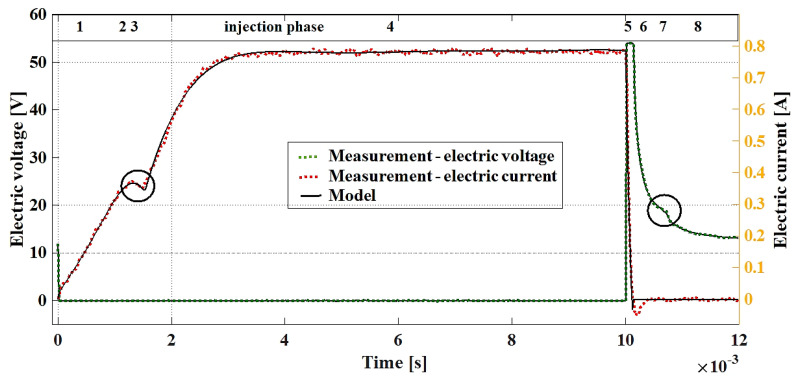
Measured and modeled electric current and voltage waveforms.

Figure 8 shows the comparison of the modeled and measured time-related current–voltage waveforms of the dosing injector, which were obtained due to the presented differential equations describing the successive ranges. The waveform that was created based on the differential equations is assigned to successive ranges of the measured waveform of the current intensity and voltage as the function of time.

## 5. Model—Supported Analysis of Injector Operation

The developed mathematical model describes the current–voltage phenomena in successive phases of the injector work. The way of describing individual sections that make up the electric current waveform of the dosing injector and the need to glue them into the continuous form results from its complicated shape and the fact of the existence of transient states. The increase in electric current and loss of voltage in the *R*−*L* circuit is described by the Kirchhoff equation. This is not true in the case where the electric circuit performing the work is considered and the derivative of the current wave changes the sign. The change of sign of the derivative of the electric current waveform can be observed at the points where the needle changes its position. This results from the work being performed by the magnetic force generated by the injector coil. When the injector needle is lifted (the beginning of the fuel injection), the magnetic permeability of the injector’s core increases ($\mu_{R}$), the magnetic resistance ($R_{m}$) decreases, inductance increases ($L_{1}$ changes to $L_{2}$), and the value of the coil time constant (τ) decreases. The conversions take place over a short time, which in consequence causes a characteristic “bend” at the needle lifting point, and this is defined by the change of sign of the derivative of the current waveform (Figure 1). Precise reflection of the function representing the current waveform, whose character varies over successive phases, by means of the differential equation, requires the usage of the coefficients inserted in the developed equations ($f_{press}$,$\;f_{k},\;f_{p}$). Due to these, the modeled electric current/voltage waveform mirrors the real conditions. 

A correctly identified model of the fuel injector in the form of an electric current waveform representing a properly working injector, after being implemented in the engine controller, allows the determination of characteristic quantities that can be treated as reference values for comparison with the actual values recorded during the injector engine operation. These quantities for the discussed injector are:

- electric current value at the point of injector core inductance increase (Figure 8; I(t)=0.35 A) and the time phase of this point (Figure 8; t=0.0014 s),
- electric current value in the steady state (Figure 8; I(t)=0.78 A),
- value of coil voltage at the inductance reduction point (Figure 8; $\left(U_{L}\left(t\right)=18\;V\right)$ and the time phase of this point (Figure 8; t=0.0016 s).

The above points on the injector current–voltage characteristics are important for controlling the injector operation, as they describe the phases of the actual fuel flow. An automatic search of these values through the engine controller (ECU) consists of observing the derivative of the current waveform in the current rise phase. The inductance increase point (spike stroke—beginning of fuel flow) determines the negative value of the derivative of this waveform. Such detection is possible after implementing elements that differentiate the observed parameter in the controller. Determining the fuel flow end point (lowering the needle to the seat and thus reducing the injector core inductance) is done to observe the derivative of the voltage waveform in the coil. After determining these points, the engine controller will have information about the characteristic values to determine the actual start and duration of the injection. These quantities can be compared with the model values obtained for current operating parameters, constituting valuable diagnostic information about the condition of the injector. 

By analyzing the behavior of the injector model and comparing the modeling results with measurements made on a real object, we could identify the phenomena occurring in the work of the injector and thus the phases of its work. The correct operation of the injector is described by a mathematical model that is defined by its coefficients. Changes in the values of the model coefficients, as well as a change in the value and time phases of the other parameters, indicate the occurrence of changes caused by damage. A set of changes or individual differences can be assigned to specific damages and determined automatically thanks to simple comparisons performed by the appropriate algorithm implemented in the engine controller. 

The model mapping of electrical phenomena occurring in the injector is very accurate; therefore, diagnostic methods based on a comparison of assumed and actual current waveforms describing the correct and incorrect functioning of the injector should also respond quickly to changes in technical condition. The possibility of detecting changes resulting from damage to the injector or fuel system based on such observations exists because the equations describing the dosing pattern of the injector contain such parameters as the core inductance, coil resistance, electromotive force, and current. Hence, we conclude that changes in current waveforms must result from the modification of any of the parameters listed. Thus, you can detect the change in the resistance of the connector or electric coil, the change in supply voltage, and the short circuit in the electrical circuit. The magnetic force that lifts the spire results from the flowing current, so this force can also be determined by observing the current waveform. The change in the form of reducing the fuel pressure in the system will be visible by reducing the value of the current at the needle’s raising point, because the magnetic flux resulting from the flowing electric current at a lower needle resistance will carry out its raising action earlier. Damage in the form of a blocked needle results in a steady increase in the electric current, without changing the derivative of the waveform, exactly as in the theoretical *R*−*L* circuit. The electrical signal, which is both the power and control signal of the injector coil, contains information about the electrical and mechanical status of the injector; therefore, observation and analysis of the waveforms allows detecting both electrical and mechanical damage to the injectors.

The method of fuel injector diagnostics carried out online by the controller based on the occurrence of differences between the model and measurement could be extremely useful for a quick diagnostics of faults. Up until now, despite the extensive on-board diagnostics (OBD II), which verifies the engine control and power supply system, some damages, especially in their the initial phase, are not precisely determined by the on-board diagnostics [33,34].

## 6. Conclusions

In this paper, a mathematical model of the injector was presented based on the analysis of the electric current and voltage waveforms in the injector circuit. The presented model is based on selecting characteristic points of the waveform, and it was verified on the test stand with the tools used in the diagnostics of the fuel injectors. The characteristics of a properly working injector were nearly identical to those of the model. Therefore, it was proved that the electric current waveform of the injector could be a valuable source of diagnostic information about the phases of injector operation and its technical state.

The article does not present a verified diagnostic method. Experiments confirming it were carried out, but they still require scrupulous development. The authors’ intention was to present a model, a tool with which new diagnostic methods can be created. These tools result directly from the differential equations described and from the explanations of physical phenomena occurring during injector dosing.

The presented considerations result from the analysis of differential equations describing the *R*−*L* circuit and principles binding the electric quantities. The structure of this article is intended to describe the tools that can be used in diagnostics and to explain the physical phenomena based on the equations presented. Individual sections of the course contain diagnostically useful information. The taxonomy described in this article allows the implementation of the presented tools in the engine controller. Inherent in the operation of fuel injectors are delays in its operation, resulting from physical and mechanical properties. The timing of the electric impulse controlling the injector needle is not identical to the resulting timing of fuel flow, which must be considered in the control. The basic information resulting from the observation of the current waveforms (Figure 4) is the possibility of determining the phase of the actual fuel flow. The engine controller (ECU) can monitor the flow phases by looking for changes in the derivative of the current waveform. Changes in the flow phases not resulting from the control indicate damage to the fuel system. By observing the flow phases, you can also determine the correctness of its functioning. This information can be used to increase the accuracy and uniformity of fuel supply.

The discussed model can be applied in an injector control system by developing the operation algorithm based on modifications within the subsequent cycles of the dosing process, the duration of the injection, and the phase of the injection, in the case when differences between the actual and model characteristics are detected [35]. Thanks to the accurate characteristics of the electric current variations, any possible differences between the model characteristics and the actual measurements for the determined control parameters will prove the existence of unwelcome modifications in the fuel system or in the injector itself. The differences, depending on their type, may be mapped onto the specific failures within the system. The algorithm is easy enough to be implemented in the engine’s ECU and will enable identification of the fuel system malfunctions in real time. Such implementation requires that the design of the control module should expanded by elements defining the derivatives of an electric current waveform with appropriate frequency that is adequate to the speed of the electric current changes taking place in the injector coil. The occurrence of discrepancies between the measurement and the model may be determined automatically, supporting the on-board diagnostics (OBD II). Then, the controller could come into action, decreasing the danger resulting from the malfunctioning injector. Thus, the function of the injector control unit will relate to the diagnostic function, due to which they complement each other, constituting a mutual-support system. Thanks to this, the quality of the control system and engine power supply will be increased. This affects the safety of the vehicle use and ensures engine operation in accordance with the requirements of ecology, extending the operation of systems dependent on the work of the injector and the engine itself.
